# Results of the national biomonitoring program show persistent iodine deficiency in Israel

**DOI:** 10.1186/s13584-022-00526-9

**Published:** 2022-03-28

**Authors:** Zohar Barnett-Itzhaki, Daniel Ehrlich, Aron M. Troen, Efrat Rorman, Luda Groismann, Moran Blaychfeld-Magnazi, Ronit Endevelt, Tamar Berman

**Affiliations:** 1 Public Health Services, Ministry of Health, 39 Yirmiyahu Street, 9446724 Jerusalem, Israel; 2 School of Engineering, Ruppin Research Group in Environmental and Social Sustainability, Ruppin Academic Center, Israel; 3 Department of Biochemistry, Food Science and Nutrition, The Robert H Smith Faculty of Agriculture, Food and Environment, The Hebrew University of Jerusalem, Jerusalem, Israel; 4 School of Public Health, University of Haifa, Haifa, Israel; 5 Department of Health Promotion, School of Public Health, Sackler Faculty of Medicine, Tel Aviv University, Tel-Aviv, Israel; 6 National Public Health Laboratory, Ministry of Health, 69 Ben Zvi Road, 6810416 Tel Aviv, Israel

**Keywords:** Human biomonitoring, Iodine, Iodine-deficiency, Desalinated water, Nutrition, Iodized salt

## Abstract

**Background:**

Adequate iodine intake is essential for human health, for normal thyroid function, and for attainment of full intellectual potential in children. In light of Israel's lack of a mandatory salt fortification policy, heavy reliance on desalination and low iodine intake from dairy products and seafood, there is concern in Israel that the population is iodine deficient. Indeed, the first Israeli National Iodine Survey in 2016 found a median urinary iodine concentration (UIC) of 83 µg/L among school age children, falling below the WHO’s adequacy range of 100–299 µg/L for children.

**Methods:**

In the framework of the National Human Biomonitoring Program in Israel, spot urine samples and questionnaire data were collected from 166 healthy children aged 4–12 years in 2020–2021. Urinary iodine concentrations were measured at the Ministry of Health National Biomonitoring Laboratory, using mass spectrometry. An international comparison of median urinary iodine concentrations (UIC) was performed taking into consideration the levels of desalinated water per capita, and fortification policies.

**Results:**

The overall median (interquartile range [IQR]) UIC was 80.1 µg/L (44.7–130.8 µg/L) indicating that the population’s iodine status has not improved in the five years that have passed since inadequacy was first identified. When comparing 13 countries with population size above 150,000, whose desalinated water per capita was at least 1 m^3^, Israel and Lebanon were the only countries with median UIC below the WHO adequacy range.

**Conclusions:**

There is an urgent need for mandatory salt fortification in Israel. Based on our international comparison, we conclude that the potential impact of desalination on iodine intake can be compensated for using the implementation of salt fortification policy. This study highlights the critical need for public health surveillance of nutritional and environmental exposures using human biomonitoring, with emphasis on vulnerable populations such as pregnant women and children.

**Supplementary Information:**

The online version contains supplementary material available at 10.1186/s13584-022-00526-9.

## Background

Iodine is an essential mineral for human health, especially as it pertains to thyroid function and neurocognitive development [1]. Iodine deficiency (ID), particularly during early childhood, may result in the development of one or multiple iodine deficiency disorders (IDD). These range from impaired thyroid function and lost intellectual potential (in mild to moderate cases), to goiter, hypothyroidism, and cretinism (in more severe cases) [2–4]. Iodine intake is especially critical for pregnant women, as fetal brain development is vulnerable to even mild iodine deficiency.

According to the World Health Organization (WHO), despite the significant risks posed by iodine deficiency, IDD can be prevented at little cost [5]. As supported by resolution WHA58.24, the preferred method of reaching and maintaining iodine adequacy on a national scale is through the adoption of universal salt iodization (USI) [6]. The basic objective of USI is to ensure that all edible salt (for both human and animal use) should be iodized [7]. Tremendous progress has been made to cope with iodine deficiency, with only 28 countries classified as iodine deficient in 2020, compared with 113 in 1993 [8, 9]. This was mostly due to effective legislation and proper enforcement of policy by countless countries around the world, all with the goal of reaching and maintaining USI [7]. Despite this global trend, Israel remains one of the last countries without any policy mandating salt fortification [10].

The first Israeli National Iodine Survey was conducted in 2016 in children and pregnant women who provided urine samples in a clinical setting. Mean urine iodine levels of school-aged children and pregnant women indicated that these populations were iodine-deficient [11]. Additional studies in recent years in Israel showed low iodine intake and low prevalence of iodized salt intake in pregnant women [12]. Previous research has shown that while Israeli milk and dairy products are iodine rich, consumption patterns of milk, dairy, as well as dairy-based foods, among Israeli adults do not ensure adequate iodine intake [13].

Studies have also raised concerns regarding the potential impact of desalinated water on thyroid health in Israel and elsewhere [14]. Israel's water supply contains over 50% desalinated water, with some areas reaching over 80%. Of note, the process of desalination not only removes salts from seawater, but also several essential minerals, most notably, calcium, magnesium, fluoride, and iodine.

The National Biomonitoring Program in Israel, established in 2020, aims to provide data on exposure of the general population to environmental chemicals including environmental tobacco smoke, heavy metals and pesticides, and on nutritional status [15]. As part of the National Biomonitoring Program, urinary iodine was measured in children and adults in 2020–2021. In the current study, we focus on findings in school aged children (SAC) as this group is used to determine population iodine status [16].

The aims of the current study were: (1) to measure urinary iodine in children and adults, (2) to evaluate the impact of consumption of certain foods (iodized salt, dairy products) on urinary iodine, and (3) to compare iodine levels in SAC in Israel and in other countries that rely on desalinated seawater, in order to test the hypothesis that the nation’s heavy reliance on desalination may contribute to Israel’s inadequate urinary iodine concentration (UIC) levels.

## Methods

### Participant recruitment

Spot urine samples for children aged 4–12 years and for adults were collected between July 2020 and June 2021 as part of the 1st cycle of the National Human Biomonitoring Program in Israel. Subjects were recruited via social media. Urine samples were collected from 166 children and 223 adults, and questionnaire data (including dietary data) were obtained via telephone interview with parents, or adult participants, by trained interviewers. Urine samples were collected on the day of the interview. The questionnaire included detailed questions on drinking water source (bottled, unfiltered tap, filtered tap), diet, consumption of iodized salt, exposure to environmental contaminants and socioeconomic status. Questions on diet and socioeconomic status were based on the questionnaire used in the National Health and Nutrition Survey in 2015–2016. The study was approved by the Tel Hashomer Hospital ethical committee (6924-20-SMC). All parents and adult participants signed informed consent forms.

### Urinary iodine measurement

UICs were measured at the Ministry of Health National Biomonitoring Laboratory. Measurement was carried out using the US CDC Inductively Coupled Plasma Mass Spectrometry Method [17], using an Agilent 7800 ICP-MS Instrument equipped with Integrated Sample Introducing System and High Matrix Introducing Mode.

Method limit of quantification (LOQ) of 2.5 μg/L was determined during method validation step by independent measurements of spiked pooled samples ("iodine-free" urine was collected for this purpose, based on previous ICP-analysis with no iodine signal detection above this in purified water blank). Accuracy (as relative recovery) and precision (as relative standard deviation) for LOQ were within 70–130% and 30% accordingly. Accuracy and precision, calculated from obtained result of purchased control material in the mid-range (20–150 μg/L) (tested with each set of samples, one CM to each ten samples) were within 96–103% and 8%.

The proficiency of the analytical procedure was established by successful participation in the CDC UIC quality assurance EQUIP [18] program and German External Quality Assessment Scheme, G-EQUAS [19].

### Statistical analysis

Wilcoxon nonparametric nonpaired tests were used to compare the urinary iodine levels between boys and girls and to compare the urinary iodine levels of children consuming specific foods/water (filtered water, bottled water, fish, cheese, milk) to those who do not consume them. These tests were used since the iodine levels were not normally distributed. Odds ratio of having a UIC in the upper vs the lower tertile in children that consumed iodized salt compared to those who didn’t consume iodized salt was calculated, and in children who consumed non-filtered water/filtered water compared to those who didn't.

### Ecological analysis

An international ecological analysis was performed comparing Israel’s median UIC level to those reported for other countries with similar levels of desalinated water per capita. In addition, fortification policies of Israel and other nations of similar desalination levels were compared, in order to examine whether USI policy might be a significant factor in determining national iodine levels. Data published in the 2020 Global Scorecard of Iodine Nutrition published by the Iodine Global Network was used [8]. This scorecard reports the most recent measurement of median urinary iodine levels (µg/L) in SAC for each nation that has such data available. Additionally, AQUASTAT database of the Food and Agriculture Organization (FAO) of the United Nations (UN) was used to obtain data on the total amount of desalinated water produced by each nation (m^3^) [20]. In order to retrieve data on desalinated water per capita, UN World Population Prospects was used to acquire data on the estimated population of each nation for the year that their desalinated water production was measured [21]. After calculating desalinated water per capita, countries in the analysis were included with per capita desalinated water intake ≥ ﻿1 m^3^ per year and with population size above 150,000. Due to some discrepancies between the year that iodine was reported and the year that desalinated water was measured, a maximum cutoff of an eight year difference was set.

An interactive map of iodine fortification published by the Global Fortification Data Exchange was used to see which countries had implemented iodine fortification policy (distinguishing between mandatory policy, voluntary policy, and no policy) [22]. Jordan was eliminated from the analysis because the reported iodine measurement was from 2010 yet the nation only implemented a salt fortification policy in 2012 [23]. For the remaining nations, median UIC was plotted versus desalinated water per capita for each country. Subsequently, sensitivity analysis was performed using only nations that were like Israel (ranked as “medium” sized) based on the criteria of both area and population combined [24]. Analyses were executed using R statistical package (version 4.0.3) and MATLAB© software (version R2019b).

## Results

86 boys and 80 girls from over 20 cities and small communities were recruited to the study, as well as 223 adults. 44 (26.5%) of the children live in rural areas and 122 live in urban areas. 71 (32%) of the adults live in rural areas and 152 live in urban areas.

The response rate in children was lower than planned due to the COVID-19 pandemic, mostly among Arabs. Therefore, only 14% of the participants (23 children) were Arab children, and not 20% (the Arab proportion in the Israeli population), as planned.

**Fig. 1:**
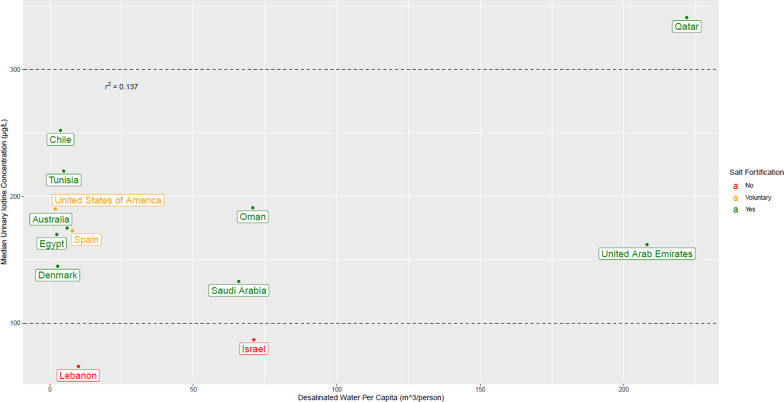
Median urinary iodine concentrations (µg/L) in children (aged 4–12 years) versus desalinated water per capita in selected countries—analysis of salt fortification policy. Dashed line indicates adequate iodine intake according to the WHO

The overall median in children (interquartile range [IQR]) UIC was 80.1 µg/L (44.7–130.8 µg/L) falling below the WHO’s iodine adequacy range for SAC of 100–199 µg/L (see Fig. 1). Of note, the mean UIC in boys (87.9 µg/L) was statistically significantly higher than in girls (63.4 µg/L, p = 0.013 (Wilcoxon nonpaired test)).

The median UIC in adults was 64.5 µg/L, even lower than that in children. Median concentrations were comparable in 120 women (62.2 µg/L) and in 103 men (70.0 µg/L) who participated in the study. Based on questionnaire data, 5.4% of the adults consumed iodized salt, while 8.5% reported not knowing whether they consume iodized salt.

Based on questionnaire data, only 14 of 166 (5.3%) children consumed iodized salt. In children with medium or high frequency milk consumption (at least one milk serving on a daily basis of low fat, above 1%, and/or chocolate milk), median UIC levels were higher than those with low frequency consumption (less than on daily basis). Particularly, the median UIC levels of medium or high frequency consumption of chocolate milk (92.9 µg/L) was statistically significantly higher than the median UIC levels of low frequency consumption of chocolate milk (60.8 µg/L, p = 0.0002). In children with medium or high frequency consumption of yellow or salty cheese (at least one cheese serving on a daily basis), median UIC level (81.4 µg/L) was statistically significantly higher than those with low frequency consumption (less than on daily basis) (55.9 µg/L, p = 0.01).

There were no statistically significant differences in median UIC in children consuming non-filtered tap, filtered or bottled drinking water or in children consuming iodized salt (see Table 1).

**Table 1:** Urinary iodine levels in children according to consumption of certain foods

Consumption frequency	N	Median	P values *
Low fat milk (0–1%)			
High	18	118.3	0.33
Medium or low	148	78.4	
Medium fat milk (> 1%)			
High	130	81.2	0.08
Medium or low	36	71.2	
Chocolate milk			
High	88	92.9	**0.0002**
Medium or low	78	60.8	
White/cream cheese			
High	126	75.4	0.31
Medium or low	40	87.2	
Yogurt			
High	92	81.8	0.86
Medium or low	74	79.6	
Yellow/salty cheese			
High	147	81.4	**0.01**
Medium or low	19	55.9	
Iodized salt **			
Yes	14	82.7	0.45
No	145	79.6	
Bottled water			
Yes	59	75.3	0.28
No	107	83.9	
Filtered water			
Yes	115	79.6	0.81
No	50	83.2	
Non-filtered water			
Yes	60	83.2	0.38
No	106	79.7	

*Nonpaired Wilcoxon test. Values in bold represent statistically significant results (p < 0.05)

**seven parents didn’t know whether their children consume iodized salt

The odds of having a UIC in the upper tertile versus the lower tertile was 1.74 times higher in children that consumed iodized salt compared to those who didn’t consume iodized salt (Odds Ratio = 1.74, 95% CI 0.39–7.68). Odds ratio for non-filtered tap water consumption compared to UIC sufficiency (upper tertile vs. lower tertile) was 1.51 (95% CI 0.68–3.34). Odds ratio for filtered tap water consumption compared to UIC (upper tertile vs. lower tertile) was 0.78 (95% CI 0.35–1.74).

When comparing all 13 countries with population size above 150,000 with desalinated water per capita was at least 1 m^3^, Israel and Lebanon were the only countries with median urine iodine levels below the WHO adequacy range (Additional file 1: Fig. S1). Israel is also the only country that did not implement a voluntary or mandatory iodine fortification policy. While Lebanon does have a policy mandating salt fortification, it has not been successfully enforced [25]. Additionally, there appeared to be low positive correlation between the amount of desalinated water per capita in each country and their median UIC levels (r^2^ = 0.132).

We also performed a sensitivity analysis that was restricted to the ten countries categorized as medium size in terms of both population and area combined [24]. Israel was found to be the only country that did not have a salt fortification policy in place. It was also the only country where median UIC levels fell below the WHO adequacy range (see Additional file 1: Fig. 1). Additionally, our sensitivity analysis indicated very low correlation between desalinated water per capita and median UIC levels (r^2^ = 0.0958).

## Discussion

The issue of iodine deficiency and the necessity of salt fortification in Israel was first raised in 2004 [26, 27]. Since then, much evidence has been collected in Israel showing low iodine intake in food, low iodine levels in desalinated drinking water, low prevalence of iodized salt consumption, and iodine deficiency in SAC and pregnant women [﻿^10^–^14^]. In recent years, there have been efforts to increase voluntary use of iodized table salt and increase awareness about this issue. Results of the Israeli National Iodine Survey in 2016, which indicated that SAC and pregnant women in Israel are iodine-deficient, were presented at several professional conferences and were reported in the media. In early 2017, the MOH published recommendations for the general public encouraging voluntary use of ionized salt [28], advanced a social media campaign, and disseminated guidelines for prenatal iodine supplement use to Ministry of Health committees and professional associations. The Israeli standard on food grade salt was updated establishing requirements for voluntary fortified salt (30 ppm iodine) and is pending final approval. Industry has also taken steps to produce cheaper iodized salt products (1 kg version) and more recently a cardboard box version; however, these are still between 2–3 times more expensive than the government price-controlled standard non-iodized salt. Recently, the Ministry of Health proposed a mandatory requirement for food fortification, including iodized salt, as part of an effort to increase public health resilience [29]. Previous studies have shown that lowered salt intake does not compromise iodine status [30]. This indicates that even if salt intake is reduced in the general population including children, according to current Ministry of Health recommendations [31], mandatory salt iodization would be sufficient to improve population iodine status.

Despite these efforts, the current study demonstrates that policy implemented in recent years by the Ministry of Health has not led to measurable improvement in the iodine status in SAC in Israel. UIC in school children remains below the WHO adequacy range. In addition, only 5.3% of children (and 5.4% of adults) in the study consume iodized salt. These results demonstrate the urgent need for mandatory table salt fortification in Israel and the clear need for increased public awareness on the importance of consuming iodized salt.

UIC surveys from SAC are the gold standard for inferring population iodine status according to WHO guidelines and there are no consensus criteria for UIC-based iodine sufficiency among adult men and non-pregnant women. Although we did not collect data on iodine status in pregnant women in the current study, it is noteworthy that median UIC for adult women (62.2 µg/L) remains like that for pregnant women in the 2016 survey (61 μg/L) [9]. This finding is consistent with a persistent adult population insufficiency and underscores the need to collect data on iodine status in pregnant women in Israel, especially in light of vulnerability of the developing fetal brain.

We cannot rule out an effect of desalination on iodine status worldwide and in Israel. However, the lack of an association in this ecological study and the iodine sufficiency of desalinating countries with iodization policy suggests that iodization can compensate for any hypothetical effect of desalination on depleting drinking water and the food chain from iodine. Of note, non-desalinated water in Israel has relatively low iodine levels (4–20 µg/L in most areas), suggesting that drinking water was not a major source of iodine in Israel even pre-desalination [32]. Since most food systems do not provide adequate iodine to support the health of the population, over 130 countries implement food and salt iodization policies as an important cost-effective, efficacious public health measure [6].

We found that children consuming dairy products (chocolate milk, yellow/ salty cheese) had higher levels of UIC. These findings are consistent with studies showing that milk and dairy are important sources of iodine in the human diet, contributing between 40 and 60% of intake [13]. Our results, while preliminary, indicate that increased consumption of milk and dairy products could contribute to higher iodine intake.

These findings also highlight the critical need for routine public health surveillance, including nutritional and environmental biomonitoring. Considering potential adverse outcomes associated with both iodine deficiency and excess iodine intake, assessing population iodine status is a key public health measure. In the US, for example, periodic and continuous monitoring of iodine levels as part of the National Health and Nutrition Examination Survey (NHANES) since 1971 has demonstrated the importance of nutritional biomonitoring to detect changes in patterns of dietary habits and nutritional content in the food supply [33]. The Canadian Health Measures Survey also includes collection of nutritional biomarkers, including folate, vitamin B12, vitamin D and iodine [34]. Indeed, when Israel introduces mandatory food fortification in the future, there will be a need for periodic and continuous nutritional biomonitoring in the population to ensure a safe, effective and sustainable fortification program. While studies aimed at evaluating the long-term consequences of iodine supplementation on thyroid autoimmunity and related dysfunction have clearly demonstrated that USI is safe, we note the importance of ongoing surveillance of thyroid function in Israel following introduction of mandatory salt iodization [35, 36].

Our study has several limitations. The most notable weakness is our small sample size. Nevertheless, the sample size is adequate to estimate a median UIC. Recruitment to the study was conducted via social media in both Hebrew and Arabic (WhatsApp messages). Recruitment of children during the COVID-19 pandemic, especially Arab children, was challenging. Consequently, it may not be accurate to generalize our findings on the iodine levels to all children residing in Israel. The current study is based on a convenience sample, including children from a range of geographic and socioeconomic backgrounds, but the sample does not necessarily represent the Israeli population. Because we recruited the sample using social media, the sample may be biased towards higher-than-average education and income populations. If so, this might have resulted in an overestimation of the true iodine intake because iodized salt use tends to be less frequent and diet quality tends to be poorer among lower socioeconomic strata [37, 38]. Finally, we did not collect data on thyroid hormone function in the study participants.

The ecological analysis relating to the use of desalinated water to median UIC data has inherent methodological limitations. In addition, we estimated crude per-capita use of desalinated water within countries and did not have data on proportional use of desalinated water for industrial and agricultural uses—factors which could influence the true exposure of the general public.

However, several strengths in our study make our findings well-suited for comparisons that may help shed light on different factors that affect median UIC. One such strength is home recruitment. Through this approach, we were able to include healthy children (unlike the first national survey that measured UIC in clinical samples) [11]. Additionally, because the age range of our cohort was like that of studies in several other countries, we were able to compare current Israeli iodine status to that of other countries. Lastly, we were able to examine the associations between dietary habits and urinary iodine concentration. Although, we were underpowered for statistical significance given the small sample size and the small number of children who used iodized salt, the OR analysis shows the association between reported iodized salt consumption and higher UIC and is consistent with a beneficial effect of iodized salt use [8].

## Conclusion

The low urinary iodine concentrations found in Israeli children indicates that the population is iodine deficient according to the WHO criteria. Moreover, our international comparisons highlight that whatever effect desalination may have on iodine status can be compensated for through the implementation of salt fortification policy. The persistence of the problem despite efforts to increase public awareness and the availability of voluntarily iodized salt, as well as campaigns on improved dietary habits, shows that voluntary measures are not enough. There is an urgent need in Israel for mandatory iodine fortification policy like that of other countries with similar reliance on desalinated seawater.

## Supplementary Information

**Additional file 1: Supplementary Fig. 1:** Median urinary iodine concentrations (µg/L) in children (aged 4–12 years) versus desalinated water per capita—analysis of salt fortification policy in ten medium sized countries. Dashed line indicates adequate iodine intake according to the WHO.

## Data Availability

The datasets generated and/or analyzed during the current study are not publicly available, but are available from the corresponding author on reasonable request.

## Abbreviations

FAO: Food and Agriculture Organization
ID: Iodine deficiency
IDD: Iodine deficiency disorders
IQR: Interquartile range
LOQ: Limit of quantification
SAC: School aged children
UIC: Urinary iodine concentration
USI: Universal salt iodization
WHO: World Health Organization
