# Supplementary material for: Results of the national biomonitoring program show persistent iodine deficiency in Israel
Source: Isr J Health Policy Res. 2022 Mar 28;11:18. doi: 10.1186/s13584-022-00526-9 (PMC8960077; doi:10.1186/s13584-022-00526-9)

**Results of the National Biomonitoring Program show Persistent Iodine Deficiency in Israel**

**Supplementary Figure 1**

Figure 1 – Median urinary iodine concentrations (µg/L) in children (aged 4-12 years) versus desalinated water per capita – analysis of salt fortification policy. Dashed line indicates adequate iodine intake according to the WHO.


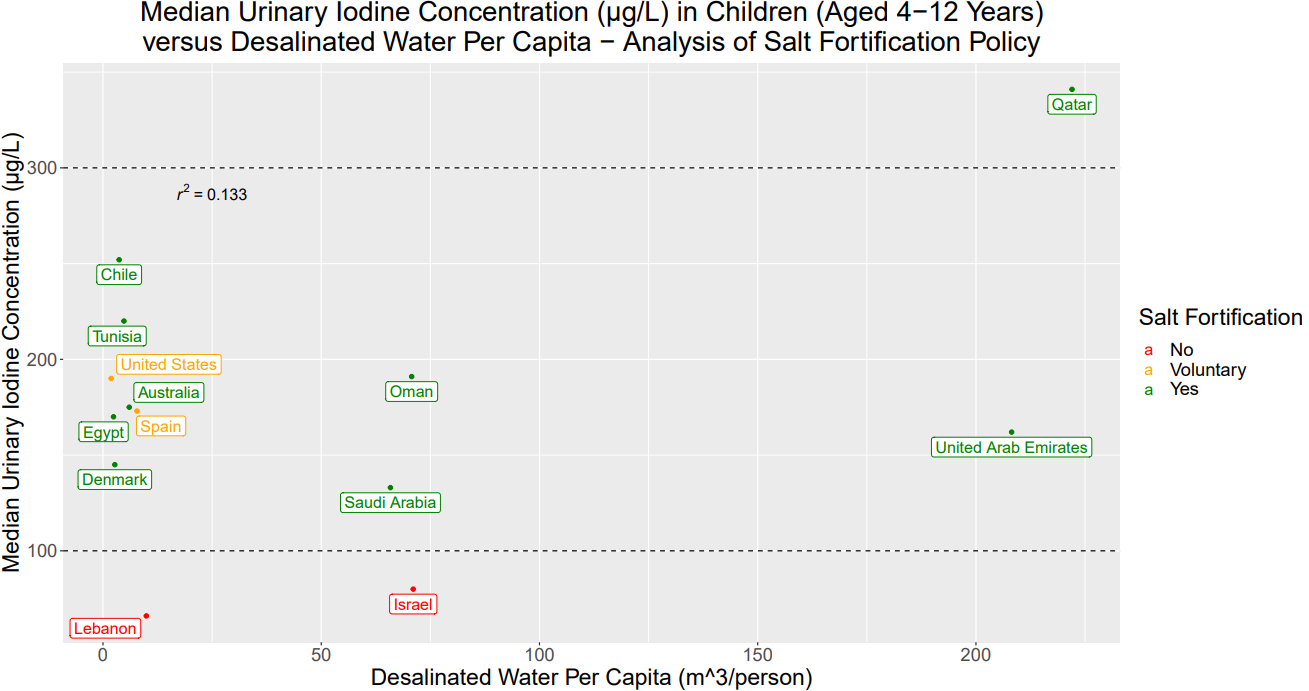

Supplement: Supplementary file 1 — Additional file 1: Supplementary Fig. 1: Median urinary iodine concentrations (µg/L) in children (aged 4–12 years) versus desalinated water per capita—analysis of salt fortification policy in ten medium sized countries. Dashed line indicates adequate iodine intake according to the WHO. [file 13584_2022_526_MOESM1_ESM.docx]
